# Path to clear conscience and how to deal with troubled conscience in older people care: a phenomenological hermeneutical study

**DOI:** 10.1186/s12912-025-03829-9

**Published:** 2025-09-15

**Authors:** Monir Mazaheri, Shima Nazari, Astrid Norberg

**Affiliations:** 1 Department of Nursing Science, Sophiahemmet University, Box 5605, Stockholm, SE 114 86 Sweden; 2 Department of Neurobiology Care Sciences and Society, Karolinska Institute, Huddinge, 14183 Sweden; 3 Lund University, Lund, Sweden; 4 Department of Nursing, Umeå University, Umeå, Sweden

**Keywords:** Clear conscience, Ethics, Nurse, Older people care, Troubled conscience

## Abstract

**Background:**

The study aimed to illuminate the meaning of conscience, and troubled conscience and how to deal with troubled conscience among nurses who take care of older people in Tehran province, Iran.

**Methods:**

A phenomenological hermeneutical approach guided the study, involving three phases of data interpretation: naïve reading, structural analysis and comprehensive interpretation. In-depth individual interviews were conducted with eight nursing staff working in nursing homes or geriatric ward in hospital.

**Results:**

The meanings of conscience, troubled conscience and dealing with troubled conscience were uncovered through three themes: “meaning of conscience”, “path to clear conscience” and “meaning of and dealing with troubled conscience”. These themes encompassed a total of eight subthemes. The nursing staff described conscience as an inherent power placed by God, shaped mainly though parent’s upbringing along with personal characteristics, religious beliefs, societal, and educational factors. Troubled conscience is narrated as inner power which blames people for intentional or unintentional mistake and attributed to themselves as they have not lived up to the type of people that they should be.

**Conclusions:**

The path to clear conscience was perceived as to do ‘right’ by helping others and to consider the others as one’s own loved one. Dealing with a troubled conscience means striving to provide right compensation. The nursing staff who take care of older people need to be supported in their endeavours to keep their conscience ‘clear’ and prevent the experience of troubled conscience.

## Background

Nursing staff (NS) often encounter ethical challenges in their daily care of patients [1], with conscience playing a pivotal role in shaping their sensitivity to the quality of care they provide [2].

Conscience is influenced by culture [3], leading to diverse interpretations across different cultural contexts. In multicultural societies, caregivers from varied cultural backgrounds, each with distinct understandings of conscience, work together to care for older people. Therefore, it is crucial to explore how these cultural variations impact both the quality of care and the well-being of NS. Divergent cultural interpretations of conscience, particularly troubled conscience, may give rise to complex challenges in caregiving. Understanding how cultural factors influence nursing staff’s conscience and their responses to ethical dilemmas in the care of older people is essential. Taking care of older people in particular, encompasses several ethical issues [4]. One of these ethical dimensions is involving conscience in care and how to deal with the troubled conscience in care of older people [5].

NS in Sweden described their conscience as a driving force, a restricting factor and a source of sensitivity [2]. NS in Iran described conscience as a supernatural, inherent power [6] with a dynamic power [7] that helps them to provide professional care [8]. Enrolled nurses (ENs) with an Iranian background, who provided care for older people in Swedish residential care settings, perceived conscience as an inner voice that should be followed directly in order to have a clear conscience [9]. According to the previous studies, the nursing staff who felt they are not able to provide a high-quality older people care, may experience troubled conscience [10, 11].

The perception of conscience has also been explored in quantitative studies conducted across various contexts. For example, Iranian NS described their conscience as an authority and asset [12], Turkish NS, as a warning signal and demanding sensitivity [13] and Swedish NS, as an authority, a warning signal, an asset, a burden, as demanding sensitivity and depending on culture [14]. These studies show that there are both similarities and variations in the understanding of conscience among NS across different countries and cultures. Perception of conscience was described as a burden, and having to deaden one’s conscience plays an important role in experiencing troubled conscience (TR) [15, 16] which may seriously jeopardize the wellbeing of NS and negatively affect the quality of their care [16]. High workload is considered a significant barrier for following conscience in performing nursing care by Iranian nurses [17], and TR was perceived as a response involving guilt, mental distress, discomfort, and unease by some nurses in Iran [18]. Meaning of having TR for the NS with Iranian background in Swedish residential care comprised as “not being a good person”, including being an uncaring people who lives in a state of unease as a result of acting against their own values [19].

Having TR was narrated by Swedish NS as ‘being trapped in powerlessness’ and ‘being inadequate’ [20]. Swedish NS in municipal older people care experienced TR when putting up with various demands, having to prioritize other assignments to being with the residents, giving poor care or not a useful care [21].

To our knowledge, no published study has explored the meaning of conscience and troubled conscience (TR) or how to address TR among nursing staff (NS) in older people care settings in Iran. Most existing studies in this field have been conducted in Scandinavian countries, highlighting the need for further research in diverse socio-cultural contexts, including countries with Muslim majorities.

This study aimed to illuminate meaning of conscience, and troubled conscience and how to deal with troubled conscience among NS who take care of older people in Tehran province.

## Methods

### Research design

The study was guided by a phenomenological hermeneutical method, which aims to disclose the meaning of lived experiences [22]. This approach involves the interpretation of data through three phases: naïve reading, structural analysis, and comprehensive interpretation [23].

The study adhered to the Standards for Reporting Qualitative Research (SRQR).

### Research context

The study was conducted in three private nursing homes and one state hospital geriatric ward in Tehran, chosen based on convenience of access and a high number of NS available at these facilities. The inclusion criteria for participants were being employed as NS in an older people care setting in Tehran and having a minimum of one year of experience in caring for older people. Once permission was obtained from the heads of these settings, the second author reached out to the nursing staff to invite them to participate in the study. The first eight people that were contacted agreed to participate. No exclusion criteria were set, and none of the invitees who accepted the invitation were excluded from the study.

The nursing homes in Iran are usually understaffed. According to the social welfare guidelines in Iran, two NS including one RN and one EN take care of approximately 50 older people with diverse health problems during the day shifts and two ENs on the evening/night shift. Weekends have lower staffing. In the hospital geriatric ward, usually three RNs (including the head nurse) and two NAs work the day shift and two RNs and two NAs on the evening/night shift, taking care of 11 older people with different diagnosis.

### Participants

A total of eight NS participated in the study, comprising three registered nurses (RNs) and five nursing assistants (NAs). The participants consisted of six women and two men, with ages ranging from 28 to 45 years. All participants worked full-time in rotational shifts. They had between 2 and 17 years of experience working as NS with older people.

### Data collection

In-depth individual interviews were conducted with the participants, during which they were asked to describe what conscience and TR meant for them. An interview guide was developed by the research team. They were also asked to narrate situations from their care of older people, from their personal lives, when they experienced untroubled versus TR, and how they dealt with TR, followed by probing questions (see interview guide, Table 1). All interviews took place in a quiet room at the participants’ workplace, with the option to choose an alternative location. The interviews, lasting between 40 and 60 min, were audio-recorded and transcribed verbatim Texts related to the meaning of conscience and TR were identified for subsequent analysis. The audio recordings and transcriptions will be securely stored for five years after the completion of the study to ensure confidentiality and may be accessed only by the research team. Since the interviewer was experienced and had conducted interviews for other studies in the same research domain, no pilot study was conducted prior to this study.

**Table 1:** Interview guide

Interview questions
What does conscience mean for you?
What does having a troubled conscience mean to you?
How do you deal with your troubled conscience?
Can you please, describe a situation form your childhood when you had a ‘clear’ conscience?
Can you please, describe a situation form your childhood when you had ‘troubled’ conscience?
Can you please, describe a situation when you experienced a ‘troubled’ conscience in taking care of older people with dementia?
Can you please, describe a situation when you experienced a ‘clear’ conscience in taking care of older people with dementia?
Can you please, describe a situation where your conscience has helped make a right decision?

### Data analysis

The data were interpreted using a phenomenological hermeneutic method [22]. The first step of interpreting the data according to Lindseth and Norberg [22] is to formulate a naïve understanding. The interviews were read several times to comprehend them as a whole. A naïve understanding regarding meanings of conscience, TR and how to deal with it were formulated. Then, the data was analysed structurally through the steps of re-reading the transcripts, finding the related data and dividing them into meaning units in line with the study aim. The meaning units were condensed and grouped based on their similarities, which were further abstracted to subthemes and themes. The interpretation was continued by going back and forth from the parts to the whole, among the themes and the naïve understanding to resolving any conflict. The process was repeated twice, from formulating a new naïve understanding followed by another structural analysis. Ultimately, a critical interpretation was written, considering the results, context, the researchers’ pre-understanding and available literature. Measures to ensure interpretive rigor were applied throughout the entire research process [24] including methodological coherence by ensuring congruence between the research question and the methods of data collection and analysis, recruiting appropriate participants with rich knowledge of the research topic, and theoretical thinking, which is apparent in the description of the analysis and results. Multiple researchers were involved in the data interpretation process. After the second author completed the naïve reading and initial analysis, the co-authors critically discussed and validated the themes and subthemes to minimize individual bias. Prolonged engagement with the data was maintained through repeated reading of the interview transcripts and reflective writing. An audit trail was kept by documenting analytic decisions, coding structures, and the development of themes. Moreover, rich and illustrative quotations from participants were included in the findings to support interpretations and provide transparency.

### Ethical consideration

The study was approved by the Organizational Joint Committee on Ethics in Biomedical Research, Tehran University of Medical Sciences, School of Nursing and Midwifery (IR.TUMS.FNM.REC.2018-067). The study carried out in accordance with the principle of the Declaration of Helsinki [25] and Swedish law (2003:460) regarding the Ethical Review of research involving human [25]. The participants were provided verbal and written information regarding the study and were assured that their participation was voluntary, with the freedom to withdraw at any time without providing an explanation or facing any consequences. Written informed consents were obtained from each participant. They were provided with contact details of the researchers in case they needed to discuss the study or experienced emotional difficulties. Confidentiality was ensured throughout the study. The transcribed audio files and the participants’ information were securely stored in a locked cabinet, accessible only to the second author. The participants’ names were replaced with pseudonyms names which was used during the analysis and reporting to the results. Careful measures were taken during data analysis and manuscript drafting to avoid disclosing any information that could potentially identify the participants.

## Results

### Naïve understanding

Meanings of conscience and TR, how they perceive developing their conscience throughout human life and how to deal with TR were narrated by the participants. Conscience is seen as an inherent power granted by God, residing in the human heart. It evolves under the influence of family, which is regarded as more influential than society, leading to variations in conscience even among people from the same family. When the NS narrated their meaning of conscience, they also elaborated on the meaning of clear conscience. NS strive to feel satisfied with themselves, especially when helping people in need, as they would a close family member, in order to achieve a calm and clear conscience.

Maintaining a clear conscience has reciprocal benefits, as it ensures that patients receive the necessary help and support. In turn, when patients are satisfied with the care they receive, it positively impacts the well-being and happiness of the nursing staff (NS) and their families, promoting a healthier and happier life for all involved. NS believed that TR is placed by God in humans to make them feel accountable for their wrong actions. Participants described experiencing TR when they intentionally or unintentionally failed to follow their conscience, resulting in feelings of a heavy heart, guilt, and shame. However, TR can also prompt them to reflect on their mistakes and seek to make amends.

Common sources of TR included providing incorrect care or care known to be wrong (e.g., fulfilling a patient’s wishes by giving sugar to diabetic older people), lack of resources, inability to prevent incorrect care by colleagues, and delegating parts of patient care to colleagues who do not perform them properly. The intensity of TR depends on the potential harm caused and the nature of the relationship with others. NS described experiencing TR despite making amends in relation to a close one, but forgetting about it in the workplace. They believed that TR negatively impacted both their present lives (e.g., insomnia, restlessness, mental preoccupation) and their future lives, as they felt that their actions would eventually return to affect them (e.g., the fate of their children, their behavior towards aging parents in need of help). To alleviate TR and achieve a sense of calm, they tried various strategies to prevent or compensate for it.

### Structural analyses

The meanings of conscience, TR and dealing with TR were revealed through three themes including “meaning of conscience”, “path to clear conscience” and “meaning of and ways of dealing with TR” and eight subthemes (Table 2).

**Table 2:** Themes and subthemes, illustrating the meaning of conscience, and troubled conscience and how to deal with troubled conscience among nursing staff who take care of older people

Themes	Subthemes
Meaning of conscience	The conscience develops throughout life
	Persons do not have the same strength of conscience
	Having conscience means to be responsible
Path to clear conscience	Clear conscience is about reaching calmness by doing right
	Keeping a clear conscience means helping others
Meaning of troubled conscience and ways of dealing with troubled conscience	An inner court blames people for intentional or unintentional wrongdoing
	Striving to prevent troubled conscience by being a good caregiver and colleague
	Dealing with a troubled conscience means to provide right compensation

### Meaning of conscience

Conscience is an inherent and inner power within humans, created by God, and shaped primarily by the manner in which parents raise their children, along with other factors such as culture, society, education, and religion. Conscience continues to develop throughout a person’s life, influenced by their cognitive abilities and experiences, guiding them to take responsibility for their actions.

The meaning of conscience illuminates in following three subthemes.

### The conscience develops throughout life

Conscience is inherent, which means that people have a conscience at time of birth. God places conscience in human beings. Being religious and faithful has impact on strength of conscience but there are also multiple other factors such as family, and society that contribute to development of conscience and its strength. The family plays a significant role in the development and strength of conscience. Conscience continues to grow with age and evolves during the life course.

Conscience evolves throughout a person’s life, continuing to grow with age. Factors such as facing life challenges and taking on greater responsibilities contribute to the strengthening of conscience, especially when these challenges are managed effectively.

*When I was a child*,* my mother had to work due to financial problems in the family*,* and I took care of my siblings. My mom really trusted me with that*,* and I think it had an effect on developing my conscience. It had a positive impact on my sensitivity to the voice of conscience at work because I took on more responsibilities*. (Nikta, 35 years old woman, working as an EN in a nursing home)

### People do not have the same strength of conscience

Considering the multiple influential factors, people have different strength of conscience. The conscience is similar all over, at time of birth. A child has a more immature conscience than adults. When a child is growing up and reaches to the puberty, conscience gets more mature.

Family members might have different understanding of what conscience is. Upbringing circumstances and ways affect the conscience. These differences further sharpened by learning from the society as well as educational system.

*I have a sister who is older than me. My sister is very cold towards my mother; she has no conscience. She has been like this since she was a child. My mother treated and loved both of us equally*,* but I don’t know why my sister has no conscience.* (Nikta, 35 years old woman, working as an EN in a nursing home)

The people’s own character, and physiological changes have also impact on conscience for example ‘cold-hearted people’ might have lower strength of conscience. A ‘cold-hearted’ might not have a strong conscience even growing up in a family with strong conscience. People themselves can act to strengthen their own conscience. People can choose to follow their conscience, hide or ignore it.

### Having conscience means to be responsible

Doing what is right is our responsibility. Having and acting in accordance with conscience means taking responsibility for our actions and their consequences.

Conscience continues to develop as we behave responsibly. One participant shared, ‘My conscience was strengthened when I tried not to make others worry or when I sought the owner of a lost item.’ (Laleh, 32-year-old woman, registered nurse caring for older people).

Taking responsibility in doing right makes one satisfied. Conscience tells us to do the right thing and avoid doing wrong. Conscience is the main actor in guiding what we do something about that and help out. “When I see that the old woman has no slippers on her feet, her feet is on cold floor without slippers, on her feet and her feet are on the cold floor, I should immediately put on her slippers or give her water when she asks for it.” (Nikta, 35 years old woman, working as an EN in a nursing home).

Taking right decisions and providing good care are not because of the older people and their families or the manager but in front of God’s eyes as the main observer.

Everyone is responsible for the conscience of their kids. It is important to take ‘clean money’ home which is the income that you have earned while doing the right things. If you do not do what you should and do not act upon your conscience, then your salary is not a clean money since you have not earned it while you were doing the right thing. Therefore, spending it on your kids impact negatively on their conscience, as one of the participants stated that “My salary that I earn by taking care of the older people, is not only for myself. I use that money to provide food and supplies not just for me, but also for my husband and children. Therefore, it must be halal (clean money), because I think of my family as well.” (Ava, 43 years woman, working as an EN in a nursing home).

### Path to clear conscience

Clear conscience is achieved when one helps a person in need, then their praying is with the person who helped. In this process, both have gained satisfaction and peace. Clear conscience has impact on current and future life of a person, because every good prayer can bring health and happiness to others´ life and to their families. The meaning of clear conscience is illustrated in following two subthemes.

### Clear conscience is about reaching calmness by doing right

Conscience can help people to choose between right and wrong. Conscience can help us to have self-control to avoid wrongdoing. One experiences clear conscience, when one gets satisfied with oneself because of doing well in helping a person in need. Anyone that can answer positively to the question ‘Do I do right things?’ experience a clear and calm conscience and peace. “Conscience is about how I do the work that I want to do, I did it right so that I can rest assured that others are satisfied with me, my conscience is clear.” (Dariush, 28 years old man, taking care of older people in a nursing home as an EN)

*One feels good when seeing that colleagues do well for the older people. A clear conscience has a positive impact on personal life*,* just as any good action for others brings positive consequences. One participant stated*,* “I owe all my life*,* my health*,* and the health of my children to the charitable prayers of the older persons I take care of*,* which is why I love them so much.* (Radmehr, 45 years old man, working as an EN in a nursing home)

### Keeping a clear conscience means helping others

Clear conscience is achieved by helping people in need. Taking care of the older people should be seen as taking care of one’s own close family member. Taking care of older people as such they take care of a close family member helps the NS to experience clear conscience. When one receives positive feedback from the older people since one has done well, one feels lightness in the heart. “I always imagine my loved ones on the older people’s side. when I visit my mother, she always says, treat the older people well, think that the older people are me (my mom).” (Nikta, 35 years old woman, working as an EN in a nursing home).

It is a mutual gain. The older people get the help they need and in turn older people’s satisfaction and comfort have a direct impact on our life and make us and our families healthy and fine. The older people pray positively for us when we help them out, then we see the results in our private life.

*When I want to avoid doing something*,* I say to myself*,* ‘Oh God*,* one day I will sleep in the same bed*,* one day I will be like this*,* I will get old… and then*,* how will it be for me?* (Faranak, 36 years old woman, working as an RN in a geriatric ward of a hospital)

### Meaning of TR and ways of dealing with TR

TR is considered as an inner power which blames people for intentional or unintentional wrongdoing. The meaning of TR defines in following three subthemes.

### An inner court blames people for intentional or unintentional wrongdoing

God placed an instinct inside human being called “Nafs Lavameh” which is developing under the influence of family. It blames people for their wrong action. TR is considered as a good sense helping people react to their intentional or unintentional mistakes. However, it could also be sensed as a psychological torture.

*It gives me a good feeling. When I’m upset or make a mistake*,* how heavy it feels*,* my heart… every time*,* I want to look back*,* but I do so with embarrassment. Every time I look*,* that mistake appears in front of my eyes*,* it’s in my heart*,* and it makes me try not to repeat it.* (Rahmer, 45 years old man taking care of older people in a nursing home, as an EN)

The common sources of TR for the NSs included having to delegate parts of the care to their colleagues who they perceived not to provide a good care, not having enough time to provide a good care for each resident, and providing less care for some resident when a NS has to focus on a specific resident, missed care, delivering the wrong care, being shy for doing the basic care, lack of resources, and inability to prevent wrong care by colleagues.

When NS are tired, both their bodies and minds could be affected. As a consequence, their conscience might get less sensitive, so they do not provide good care. Later, when they take a rest, they review their actions and might experience TR.

### Striving to prevent TR by being a good caregiver and colleague

One should try not to create situations that might cause a TR. Some means are to check the care activities and older people’s conditions with colleagues more often, try to study oneself and stay up to date on care methods and how to get the best results for older people.

It is also important not to care about other persons´ reflections when NS confess their wrongdoing. The previous mistakes can be learnt as lessons that help to prevent experiencing TR in similar cases. For instance, one of the participants stated that:

*The patient did not know*,* but I knew that I made a mistake. However*,* she lost her trust in me; she was admitted to our ward two more times. Maybe she thought about me differently*,* but I was calm because I told myself*,* yes*,* I gained an experience. I pay attention to orders to avoid repeating that mistake.* (Tahmineh, 38 years old woman, working as an RN in a geriatric ward of a hospital)

Taking vacation and enough rest between the working shifts also helps to prevent situations that create TR. Establishing close relationship with the older people can also help. The good working relationship with the colleagues is also important in providing a good care that brings less risk of TR. A friendly working culture helps to keep the caregiver’s conscience awake.

Sometimes, one should provide the best care for the older people even if it does not make them very satisfied for example regarding the diet restrictions for an older person with diabetes.

*I went and sat close to him and said*,* please forgive me*,* I explained the reason. It (sugar) is harmful for you*,* you should not drink tea with sugar a lot. You should not*,* I said it like this*,* then he said*,* it does not matter.* (Radmehr, 45 years old man, working as an EN in a nursing home)

Some colleagues might need an alarm to be aware of their conscience and do the right thing. The advice has impact on colleagues’ behaviours. The colleagues should be warned if they do not provide a proper care, so they can get supervision so that they can be monitored to do the right thing.

Managers have an important role in preventing TR for example by encouragement, providing guidance and motivating the NS to work with attention or supporting the older people. They also need to consider reasonable working loads and not planning very long working shifts like 24–48 h.

### Dealing with a TR means striving to provide right compensation

When one causes discomfort or problems for the older people, one should compensate that harm as soon as possible regardless of the consequences for oneself in order to ease the TR. The compensation can be in form of explaining the incidence to the people for example if one has missed to shower the older people, apologizing, and helping them to get shower and wear new clothes. The compensation calms one down, afterwards, if the older people become satisfied with the NS. It is important to compensate with showing love and affection, hugging, and kissing them. Compensation is not only about the older people but also in relation to mistakes one does in communicating with the colleagues or during common activities. It is also important to compensate for the colleagues’ mistakes and for the older people that were affected by that mistake. If one becomes aware of a mistake regardless of if who has made it, one must try to make it up for the patients, without any expectations from the wrongdoer, but showing understanding.

It is possible to put the memory of TR behind and move forward if the mistake gets compensated. However, there is a difference if the issue is at work regarding one of the older people or at home in relation to a family member or relative. The older people get discharged and one does not see them anymore, so it is easier to move forward and forget the bad memory of TR, but one continues to meet the family members and relatives, so the sense of TR continues to live with one.

*If I injected the wrong medicine*,* either to my sister or to another patient*,* I have the same troubled conscience. I look to see the degree of damage*,* but because a patient stays with us for a short time*,* and my mistake is related to my work*,* I can forget it after compensation. But in my family relationship*,* we see each other daily*,* so I cannot forget my mistake easily even after compensation*,* and it takes longer.* (Tahmineh, 38 years old woman, working as an RN in a geriatric ward of a hospital)

We have much higher priority in satisfying our residents than our managers. It is the big love for the residents that is our main drive.

*My priority is the patient. I have to satisfy the patient*,* and the management has to satisfy me. I cannot take care of the patient just for the satisfaction of the management and do three or four other things instead.* (Ava, 43 years old woman, working as an EN in a nursing home)

### Critical interpretation and reflections

The meaning of conscience was narrated as an inherent power placed by God inside human beings and influenced mainly by parents’ upbringing along with personal characteristics, cultural, societal, and educational factors. Only few studies conducted in Western countries stated conscience as an instinct [26–28]. However, studies among Iranian NS [9, 19] confirm the results. Conscience is narrated by Iranian NS as the vigilant, blaming and moral agent [29]. Jasemi, Aazami et al. (2019) concluded that religious beliefs can help NS to provide a care in-line with the conscience by reinforcement of their professional commitment and help them to avoid ignoring older people’s needs [17].

In this study, conscience is described as the main actor in guiding people to do the right thing and avoid mistakes, therefore they demanded people to follow their conscience directly. The results are similar to findings of studies among Iranian NS in Iran and Sweden [9, 30]. In the Middle Ages, philosophers in Europe meant that the conscience can be wrong [31], as also was stated in a study on Swedish healthcare staff [14]. Ericson-Lidman and Strandberg (2013, 2015) emphasized NSs´ need to listen and interpret the voice of conscience by themselves and together with colleagues for sharing knowledge rather than mere following conscience [21].

The NS narrated that they could reach clear conscience by doing right and providing good care to older people in need as such they provide care to their close family member. In Buddhism, Confucianism, Hinduism, Islam, Judaism and Christianity, for example, the Golden Rule is invoked: “Whatever you want people to do to you, do to them” [32]. The participants wanted to treat the residents as their old family members. This is a mutual advantage that leads to the older people´s satisfaction and comfort which have a direct impact on NS’s personal and working life. NS with Iranian background in Sweden, believed that clear conscience is associated to their relationships with the older people, therefore, they tried to provide affectionate care and to treat them as they expect to be treated [9]. NS in another study in Iran shared their feeling of living with the older people as family members and felt responsible for them [33] which seems to be common on studies on people with Iranian background in different contexts.

TR is described by the NS as an inner power, blaming for mistakes. Being unable to follow the conscience may generate the sense of guilt, sadness, hopelessness, and powerlessness among nurses [34]. Feeling of being unable to help people in need can lead to feeling of TR [35], guilt for not providing suitable care [33] and shame [19]. NS in Sweden described themselves primarily not being guilty because of doing wrong but as not being “adequate” because they could not do what they felt they should. Thus, shame of not being ‘good enough’ rather than guilty emerged primarily as a TR in them [36]. Another Iranian study reported that NS were constantly dwelling in their memories of their mistakes despite of compensation, however, they described their experience as an opportunity to rebuild their relationship with God and seek his help [37]. Swedish NS described their TR was due to factors outside themselves, as a result of being caught between the conflicting demands of their residents, the residents’ relatives, colleagues and their own sometimes conflicting demands [33], deficient social support from their superiors [38] and colleagues [16]. These findings indicate that NS in Sweden often did not accuse themselves of causing their TR. However Iranian NS blame themselves for their mistakes and try to compensate to become relieved.

In this study, common sources of TR were lack of confidence in colleagues to entrust some of the care activities, time shortage, providing wrong care, being shy of providing the basic care, lack of resources and inability to prevent colleagues’ wrong care. The heaving workload and low staffing in nursing homes in Iran can contribute intensify the situation. Form a good relationship with the older people and their families and colleagues, rechecking the provided care with colleagues, allocating enough time to each older people, creating a happy atmosphere at work, keeping up to date and taking a rest, were considered by NS as ways for preventing TR. NS emphasized the importance of compensation for their mistakes as soon as possible regardless of the consequences in comforting the TR.

Ethical issues arose when choosing between acting in one resident’s best interests and preventing other residents from coming to harm [39]. Work overload and lack of resources are barrier for performing nursing care in line with nurses´ or care providers´ conscience [17], for providing compassionate nursing care [40] and unconscionable acts [6]. The conscience needs to be informed and educated with help from others [21]. Iranian nurses described forming closer relationships with older people, following one’s moral beliefs and principles, attempting to achieve inner peace by sympathy with older people, satisfying their needs to please God, visiting older people regularly as strategies to go beyond the routine nursing care [41]. The Iranian ENs in Sweden tried to compensate their TR by providing better care to the older people [19]. Swedish NS experienced TR when putting up with various demands, having to prioritize other assignments to being with the residents, giving poor care or not a useful care [21].

Taking rest was a strategy for the participants to reduce TR and keep being sensitive to their voice of conscience. Distancing and energizing for emptying heads and recharging batteries through lying down and relaxing and resting after work were shown as a way of dealing with TR in a Swedish study as well [42].

Our participants believed that managers have an important role in preventing TR for example by encouragement, providing guidance and motivating the NS to work with attention or supporting the older people. A poor social support from superiors but not from colleagues and family/friends has been shown the main source of stress of conscience [38].

The negative experience of TR motivates nurses to change their behaviour to prevent errors and lead to positive changes that improve patient safety and promote accountability. NS should be enabled to develop skills and knowledge regarding ethical reasoning, critical self-awareness and effective interpersonal communication strategies through their education and into practice to assist in issues of conscience. If nurses are enabled to share their TR with others, then constructive ways to approach and deal with this can be developed [43].

The study calls for further exploration of meaning of conscience, troubled conscience and managing it among nursing staff in diverse cultural contexts to gain a broader understanding of the concepts, identifying the factors that might lead to a troubled conscience and how to manage it. In current multicultural societies, people with varied cultural backgrounds and different understanding of conscience work together as caregivers. Therefore, it is essential to learn about the variation of such understanding and how it impacts the quality of care and also the nursing staff’s wellbeing.

Divergent cultural interpretations of conscience and troubled conscience between caregivers and care recipients may lead to challenging scenarios in care settings. Therefore, we need to know how cultural aspects may influence nursing staff’s conscience and their response to ethical challenges in care of older people.

### Study limitation

Considering the qualitative nature of the study in particular as a phenomenological hermeneutical study, demanding few participants, the findings can not to be generalized to whole population of nursing staff working in older people care settings. However, the findings can be transferred to the similar contexts and settings.

Most of the nursing staff working at nursing homes in Iran are women [44], which reflect the gender distribution of the participants in this study. Gender perspectives might have some impact on understanding of the phenomenon of interest and need to be consider in further studies.

Another limitation of the study considering interviews as the data collection method is that the participants may find it difficult to share their true experiences and reflections with the researchers. However, we tried to decrease this risk by choosing the settings that none of the researchers have worked there previously or knew any of their working staff in person. The researcher who conducted the interviews, make all attempts to provide a neutral supportive environment for the participants to share their experience. The participants were ensured that no one else than the interviewer know their identity and their personal information will be removed and not shared with anyone including the rest of research team.

### Implications for practice

Nursing staff’s strategies in dealing with their troubled conscience in care of older people varies and their cultural backgrounds need to be considered. The current study give insight about the subject in a specific cultural context. Insight to the sources of troubled conscience for the nursing staff is valuable in planning strategies to prevent troubled conscience among them. Sources included lack of confidence in colleagues to delegate some of the care activities, time shortage, providing wrong care, being shy of providing the basic care, lack of resources and inability to prevent colleagues’ wrong care.

The strategies of the nursing staff in this study to prevent experiencing troubled conscience were developing a good relationship with the older people, their families and colleagues, rechecking the provided care with colleagues, allocating enough time to each older people, creating a happy atmosphere at work, keeping up to date and taking a rest. Such strategies might be valuable for other nursing staff as well.

The nursing staff in this study emphasized the importance of compensation for their mistakes as soon as possible and regardless of the consequences in order to comfort their troubled conscience. The nursing staff should be supported in opening up and in their attempts to compensate.

Taking rest was a strategy for the participants to reduce troubled conscience and keep being sensitive to their voice of conscience. Distancing and getting rest as a way of dealing with troubled conscience should be encouraged among nursing staff.

Appropriate policymaking should support the nursing staff in their endeavours to keep their conscience ‘clear’ and prevent them having troubled conscience. Such supports in its turn affect nursing staff’s wellbeing and quality of the care they provide.

The nursing staff who take care of older people should be enabled to develop skills and knowledge regarding ethical reasoning, critical self-awareness and effective interpersonal communication strategies through their education and into practice to assist in issues of conscience. Such competence should be developed in their education.

Our participants believed that managers have an important role in preventing troubled conscience for example by encouragement, providing guidance and motivating the nursing staff to work with attention or supporting the older people. A poor social support from superiors but not from colleagues and family/friends contribute to dissolved troubled conscience and distress among nursing staff. The literature demonstrated that the ethical climates at working environment has impact on professional practice including providing individualized care for older people, in older people care settings and the [45]. Suhonen et al. [38] state that organizational context of care settings influences the provision of nursing care, emphasizing the need for nursing leaders to investigate the ethical climate and implement measures for improvement. The diversity of understanding conscience should be considered in designing research studies and educational contents.

## Conclusion

The NS described conscience as an inherent power placed by God in human beings, influenced primarily by parents’ upbringing, along with personal characteristics, religious beliefs, societal norms, and educational factors. Troubled conscience (TR) is narrated as an inner force that blames people for intentional or unintentional mistakes, reflecting a failure to live up to the type of person they believe they should be.

The path to a clear conscience is achieved through doing ‘right’, by helping others and considering others as one’s own loved ones. NS need support in their efforts to keep their conscience calm and prevent troubled conscience. However, the findings should be interpreted with consideration of the cultural context.

Understanding how NS manage troubled conscience and how they perceive conscience in general can help nursing managers provide appropriate support, enabling staff to handle troubled conscience and mitigate situations that may trigger it. This, in turn, contributes to delivering high-quality care to patients while safeguarding the well-being of the NS.

## Data Availability

The datasets generated and/or analyzed during the current study are not publicly available to uphold informed consent and maintain confidentiality but can be obtained from the corresponding author upon reasonable request.

## Abbreviations

Ens: Enrolled nurses
NS: Nursing staff
TR: Troubled conscience
